# Liposomal bupivacaine versus conventional anesthetic or placebo for hemorrhoidectomy: a systematic review and meta-analysis

**DOI:** 10.1007/s10151-023-02881-4

**Published:** 2024-01-31

**Authors:** P. Solis-Pazmino, L. Figueroa, K. La, O. Termeie, K. Oka, M. Schleicher, J. Cohen, M. Barnajian, Y. Nasseri

**Affiliations:** 1 Surgery Group Los Angeles, Los Angeles, CA USA; 2 Surgery Department, Santa Casa de Porto Alegre, Porto Alegre, RS Brazil; 3 Knowledge and Evaluation Research Unit, Mayo Clinic, Rochester, MN USA; 4 CaTaLiNA-Cancer de Tiroides en Latino América, Quito, Ecuador; 5 Facultad de Ciencias Médicas, Universidad Central del Ecuador, Quito, Ecuador; 6 Cleveland Clinic, Cleveland, OH USA; 7 Cedars-Sinai Medical Center, Los Angeles, CA USA

**Keywords:** Liposomal bupivacaine, Postoperative pain, Hemorrhoidectomy, Postsurgical pain

## Abstract

**Background:**

Liposome bupivacaine (LB) is a long-acting anesthetic to enhance postoperative analgesia. Studies evaluating the efficacy of the LB against an active comparator (bupivacaine or placebo) on acute postoperative pain control in hemorrhoidectomy procedures are few and heterogeneous. Therefore, we performed a systematic review and meta-analysis comparing LB’s analgesic efficacy and side effects to conventional/placebo anesthetic in hemorrhoidectomy patients.

**Methods:**

We performed a systematic review and meta-analysis of randomised controlled trials investigating the use of LB after haemorrhoidectomy. We searched the literature published from the time of inception of the datasets to August 19, 2022. The electronic databases included English publications in Ovid MEDLINE In-Process & Other Non-Indexed Citations, Ovid MEDLINE, Ovid EMBASE, and Scopus.

**Results:**

A total of 338 patients who underwent a hemorrhoidectomy procedure enrolled in three randomized clinical trials were included. The overall mean age was 45.84 years (SD ± 11.43), and there was a male predominance (53.55% male). In total 194 patients (52.2%) received LB and 144 (47.8%) received either bupivacaine or placebo. Pain scores at 72 h in the LB (199, 266, and 300 mg) were significantly lower than in the bupivacaine HCl group (*p* = 0.002). Compared to the bupivacaine/placebo group, the time to first use of opioids in the LB group was significantly longer at LB 199 mg (11 h vs. 9 h), LB 266 mg (19 h vs. 9 h), and LB 300 mg (19 h vs. 8 h) (*p* < 0.05). Moreover, compared to the bupivacaine/epinephrine group, it was significantly lower in the LB 266 mg group (3.7 vs. 10.2 mg) and at LB 300 mg (13 vs. 33 mg) (*p* < 0.05). Finally, regarding adverse effects, the conventional anesthetic/placebo group reported more pain in bowel movement than LB groups (OR 2.60, 95% CI 1.31–5.16).

**Conclusions:**

Comparing LB to conventional anesthetic/placebo anesthetic for hemorrhoidectomy, we found a statistically significant reduction in pain through 72 h, decreased opioid requirements, and delayed time to first opioid use. Moreover, the conventional anesthetic/placebo group reported more pain in bowel movement than LB groups.

**Supplementary Information:**

The online version contains supplementary material available at 10.1007/s10151-023-02881-4.

## Introduction

An excisional hemorrhoidectomy is a successful approach for patients who fail conservative treatments [1]. Still, it is associated with significant postsurgical localized pain resulting from the surgical incision of the sensitive and highly innervated anoderm and anal mucosa [2]. Therefore, it increases the need for additional analgesics and postpones the return to social life and work.

An effective local analgesic that offers prolonged pain relief beyond the capabilities of current agents would present a valuable therapeutic choice for effectively managing postsurgical pain. Liposome bupivacaine (LB) is a long-acting anesthetic used for postoperative analgesia. This medication has microscopic, spherical, and multi-vesicular liposomes that augment the local anesthetic action time via delayed liposome release and slow the peak plasma concentration compared to typical bupivacaine. Its effects last up to 72 h [3].

LB has been explored in several fields, suggesting advantages over conventional agents [4, 5]. Its role in hemorrhoidectomy has recently been reported. Studies evaluating the efficacy of the LB against an active comparator (bupivacaine or placebo) on acute postoperative pain control in hemorrhoidectomy procedures are few and heterogeneous. Therefore, we sought to perform a systemic review and meta-analysis comparing the analgesic efficacy at 72 h, the time to first opioid, the dose of rescue medication over 72 h, and adverse effects (AE) in hemorrhoidectomy patients.

## Methods

We followed the Preferred Reporting Items for Systematic Reviews and Meta-analysis (PRISMA) guidelines.

### Eligibility criteria

We included randomized, double-blind, placebo-controlled clinical trials (RCTs) in men and nonpregnant women aged 18 years or older scheduled to undergo excisional hemorrhoidectomy. Patients were excluded if they received less than 133 mg LB (previous studies have not demonstrated efficacy with lower doses) [6, 7]. Additionally, we excluded patients who took analgesics (non-steroidal anti-inflammatory drugs, acetaminophen, or opioids), antidepressants, or glucocorticoids within the 3 days before surgery. Studies with fewer than 20 participants were excluded.

The primary outcome of interest was a pain score at 72 h. Secondary outcomes were time to first opioid, the dose of rescue medication over 72 h, and adverse effects (AE).

### Data sources and searches

A comprehensive search of several databases was conducted from inception until August 2022. The databases included were Ovid MEDLINE and Epub Ahead of Print, CINAHL, Ovid EMBASE, Ovid Cochrane Central Register of Controlled Trials, Ovid Cochrane Database of Systematic Reviews, Scopus, and Web of Science—searched Science Citation Index (SCI), Conference Proceedings Citation Index (CPCI), and BIOSIS Citation Index (BCI). The search strategy was designed and conducted by an experienced librarian with input from the study’s principal investigator. Controlled vocabulary supplemented with keywords was used to search for studies of liposomal bupivacaine for hemorrhoidectomy (Supplementary eMethods https://github.com/NotLui5/liposomal-bupivacaine_others).

### Study selection

Search records were uploaded into Covidence systematic review software, Veritas Health Innovation, Melbourne, Australia. All stages of the review (title and abstract screen, full-text screen, and data extraction) were duplicated by three independent reviewers (PS-P, KO, KL, LF). Before beginning each stage, pilots were performed to understand and accurately understand the eligibility criteria. Disagreements at each stage of the review were resolved by the senior author (YN). Full-text screening agreement was assessed using Cohen’s kappa (κ = 0.85).

### Data collection

The following data were extracted: (1) general characteristics (first author, publication date, country, study design, data collection period); (2) setting (single-center, multicenter); (3) preoperative characteristics (age and sex); (4) primary outcomes (pain relief) was assessed by the cumulative pain score as reflected in the pain intensity at rest measured using a validated 10-point numeric rating scale (NRS; 0 = no pain and 10 = worst possible pain) area under the curve through 72 h after study drug administration (AUC 0–72).

Secondary outcomes were the total amount (milligrams) of opioid rescue medication consumed within 72 h after surgery, the time to first postsurgical use of opioid rescue medication, and adverse effects (AE). Adverse effects were defined as any AE occurring after administration of the study drug. All AEs were classified by system organ class and summarized by treatment group.

### Risk of bias assessment

Study quality was assessed by three independent reviewers (PS-P, KO, KL). Disagreements were resolved through consensus by including two reviewers (YN, PS-P). To determine the risk of bias in RCTs, we used the RoB2 Cochrane tool. The domains of this tool are (1) the randomization process; (2) deviations from intended interventions; (3) missing outcome data; (4) measurement of the outcome; and (5) selection of the reported result. Each question had four possible responses: “yes,” “probably yes,” “probably no,” “no,” and “no information.” For a better understanding, “definitively yes” was interpreted as a low risk of bias, “probably yes” and “probably no” as unclear, and “definitively no” as a high risk of bias.

The overall risk of bias was calculated on the basis of the responses to each of the five domains. Studies with at least one domain considered as a “high risk of bias” or with multiple domains considered as “some concerns” in a way that substantially lowers confidence in the result were judged to be at a high overall risk of bias; studies with at least one domain at “some concerns” were considered to be at some concerns the overall risk of bias. Those studies with all domains classified as “low risk of bias” without any “some concerns” or “high risk of bias” domains were at a low overall risk of bias. This approach has been used before.

### Certainty in the body of evidence

The quality or certainty of the evidence was assessed with the Grading of Recommendations Assessment, Development, and Evaluation (GRADE) approach [8]. This assessment reflects the confidence level that the effect sizes or estimates from this systematic review and meta-analysis are correct.

Working individually, one reviewer (PS-P) assessed the quality of evidence, and disagreements were resolved by consensus involving a second reviewer (YN). Overall, the quality of the evidence of each treatment-comparison-outcome triad can be graded as very low, low, moderate, and high. To assign these, we began by rating randomized trials as high-quality and observational studies as low-quality evidence. Then, on the basis of different factors, we either downgraded (risk of bias, inconsistency, indirectness, imprecision, and publication bias) or upgraded (large magnitude of effect, plausible confounding, and dose–response gradient) the initial rating.

### Statistical analyses

We calculated each study’s odd ratio (OR) and 95% confidence interval (CI) using an intention-to-treat analysis approach for dichotomous outcomes. Continuous variables, such as pain score and opioid requirement, were expressed as mean and standard deviation. Fisher’s exact test was used to compare between-group differences.

We used RStudio, an integrated development environment for R [9], to perform the analyses and generate forest plots. Heterogeneity across studies was assessed with a study variance estimate (tau squared). The proportion of variability in effect size estimates attributed to between-study heterogeneity was assessed with the *I*^2^ statistic [10].

## Results

### Search results

**Fig. 1:**
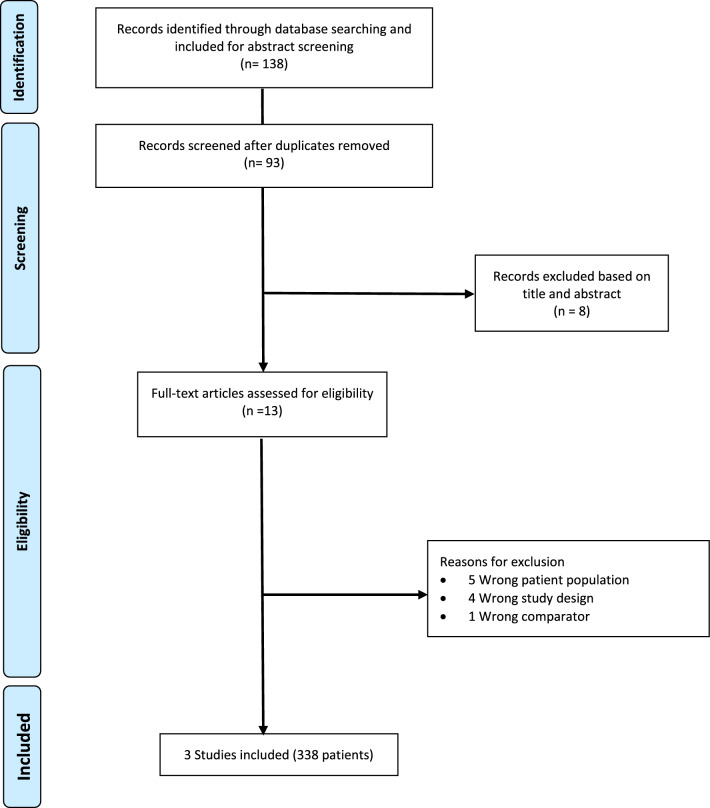
PRISMA flow diagram of the study selection process

The initial literature search generated a total of 138 studies. After deduplication and screening, three RCTs met our inclusion criteria (Fig. 1).

### Study characteristics

Three studies [11–13] were randomized, double-blind, and placebo-controlled (Table 1). The studies were published from 2011 to 2012. The overall risk of bias was high in one study and with some concern in two studies (Supplementary eFig. 1 https://github.com/NotLui5/liposomal-bupivacaine_others). Two were multicenter studies [11, 13] (conducted in more than nine centers). All were conducted in the USA.

**Table 1:** Baseline characteristics

Author, year	Country	Study design	Groups		Population number	Age (years, mean ± SD)	Female (*n*)	Male (*n*)
Miller et al., 2011 [12]	USA	Randomized clinical trial	Intervention	LB 225 mg	25	NR	NR	NR
				LB 300 mg	25			
			Comparison	Bupivacaine/epi 75 mg	25			
Gorfine et al., 2011 [13]	USA	Randomized clinical trial Multicenter	Intervention	LB 300 mg	94	48 (12.2)	32 (34%)	62 (66%)
			Comparison	Normal saline	93	48.7 (11.9)	27 (29%)	66 (71%)
Haas et al., 2012 [11]	USA	Randomized clinical trial Multicenter	Intervention	LB 199 mg	25	42 (11)	9 (36%)	16 (64%)
				LB 266 mg	25	46 (11)	3 (12%)	22 (88%)
			Comparison	Bupivacaine HCl 75 mg with epinephrine	26	44 (11)	11 (25%)	15 (75%)
Total			Intervention	LB	194	45.33 (11.40)	44 (23%)	100 (77%)
			Comparison	Other	144	46.35 (11.45)	38 (26.4%)	81 (73.6%)

### Patient characteristics

Patient demographics and preoperative variables are outlined in Table 1. In total, 338 patients were included; 194 received LB at four different doses (119 at LB 300 mg, 25 at LB 199 mg, 25 at LB 225 mg, 25 at LB 266 mg) and 144 received control medication (51 bupivacaine/epinephrine and 93 normal saline). The overall mean age was 45.8 years (SD ± 11.4), and there was a male predominance in the overall cohort (181 [53.5%] men vs. 82 [46.5%] women).

### Primary outcome

#### Pain scores

Two studies reported pain intensity, as reflected by the mean cumulative pain scores (AUC of NRS). Table 2 showed that pain scores at 72 h in the LB 199 mg and 266 mg groups were significantly lower (AUC 180) than in the bupivacaine HCl group (AUC 340, *p* = 0.002) [11, 13]. Additionally, one study reported that pain was considerably less in LB 300 mg compared with placebo (0.9% sodium chloride) (AUC 141.8 vs. 202.5, *p* < 0.0001) [11, 13].

**Table 2:** Postoperative pain score through 72 h after administration of the drug

Author, year	Intervention	Population number	AUC mean (SD)^#^	Mean difference	*p* value
Gorfine et al., 2011 [13]	LB 300 mg	94	141.8 (10.7)	60.7 (58–64)	< 0.0001
	0.9% sodium chloride	93	202.5 (10.7)		
Haas et al., 2012 [11]	LB 199 mg	25	180 (NR)	160 (154–166)	0.002
	LB 266 mg	25	180 (NR)		
	Bupivacaine HCl 75 mg with epinephrine	26	340 (NR)		

*AUC* area under the curve, *NRS* numeric rating scale

^#^Pain relief assessed with the cumulative pain score as reflected in the NRS area under the curve through 72 h after study drug administration (AUC0–72)

*Pain relief assessed with the visual analog scale

### Secondary outcomes

#### Time to the first opioid

Three studies reported the median time to the first use of opioid rescue medication after surgery. Compared to the bupivacaine/placebo group, the time to first use of opioids in the LB group was significantly longer at LB 199 mg (11 h vs. 9 h), LB 266 mg (19 h vs. 9 h), and LB 300 mg (19 h vs. 8 h) (*p* < 0.05) [11–13]. Moreover, the time to opioid rescue in LB at 300 mg was longer than normal saline (14 h 20 min vs. 1 h 10 min, *p* < 0.0001) [11–13].

#### Dose of opioid rescue medication

Three studies report this outcome. In the LB groups, 66 (70.2%) patients had opioid rescue medication and 28 (29.8%) had none. In the placebo group, 84 (90%) patients had opioid rescue medication and 9 (10%) did not. At 72 h after the study drug administration, the mean total amount of opioid rescue medication (morphine equivalents) consumed was significantly lower in the LB 300 mg group compared to normal saline (22.3 vs. 29.1 mg; *p* < 0.0006) [11–13]. Moreover, compared to the bupivacaine/epinephrine group, it was significantly lower in the LB 266 mg group (3.7 vs. 10.2 mg) and in the LB 300 mg group (13 vs. 33 mg) (*p* < 0.05) [11, 12].

#### Adverse effects (AE)

**Fig. 2:**
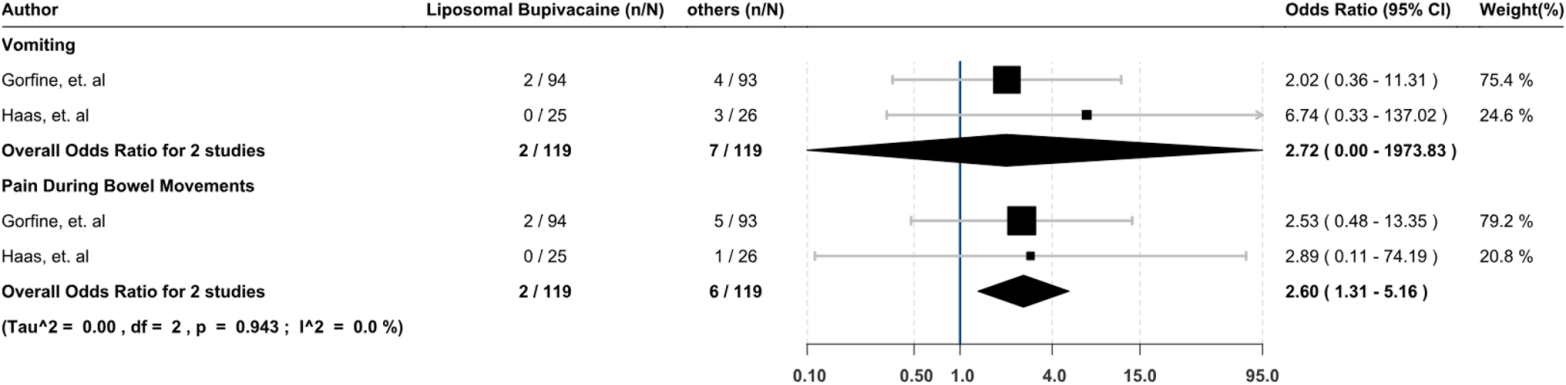
Adverse effects

Two studies reported on AE. Regarding vomiting, our meta-analysis revealed no difference in LB compared to conventional anesthetic or normal saline groups (OR 2.72, 95% CI 0.00–1973.83). However, both conventional anesthetic and normal saline groups reported more pain in bowel movement than LB groups (OR 2.60, 95% CI 1.31–5.16) [11, 13] (Fig. 2).

### GRADE approach to assess the quality of evidence

Overall, the quality of evidence was very low for all comparisons (Table e-6). The quality of evidence was downgraded for imprecision (very small information size, very few events, and 95% CI overlaps with no effect). Indirectness was not an issue because all studies directly compared the interventions we are interested in, delivered to the populations we are interested in, and measured the outcomes important to patients (Table 3).

**Table 3:** Time to the first opioid

Author, year	Intervention	Population number	Median (IQR)	*p* value
Miller et al., 2009 [12]	LB 300	25	19 h	< 0.05
	Bupivacaine/epi 75 mg	25	8 h	
Gorfine et al., 2011 [13]	LB 300 mg	94	14 h 20 min	< 0.0001
	0.9% sodium chloride	93	1 h 10 min	
Haas et al., 2012 [11]	LB 199 mg	25	11 h (0.2–96.0)	0.0005
	LB 266 mg	25	19 h (0.2–96.0)	
	Bupivacaine HCl 75 mg with epinephrine	26	9 (0.1–96.0)	

## Discussion

To our knowledge, this is the first systematic review and meta-analysis evaluating the effects of LB on pain for patients undergoing hemorrhoidectomy. We found that LB was associated with decreased pain score at 72 h, longer time to first opioid use, decreased opioid use postoperatively, and comparable adverse effects to control or placebo (Table 4).

**Table 4:** Dose of opioid rescue medication (mg) in 72 h

Author, year	Intervention	Population number	Mean mg (SD)	*p* value
Miller et al., 2009 [12]	LB 300	25	13	< 0.05
	Bupivacaine/epi 75 mg	25	33	
Gorfine et al., 2011 [13]	LB 300 mg	94	22.3 (21)	< 0.0006
	0.9% sodium chloride	93	29.1 (20.7)	
Haas et al., 2012 [11]	LB 266 mg	25	3.7 (NR)	0.0019
	Bupivacaine HCl 75 mg with epinephrine	26	10.2 (NR)	

Although LB was approved by the US Food and Drug Administration (FDA) in 2011, its role in colorectal surgery is only relatively recently reported. In 2018, Raman et al. [14], in a meta-analysis including seven high-risk bias studies (*n* = 1008) in patients undergoing laparoscopic or open colectomy, reported that pain scores were significantly lower in patients who received LB (local or transversus abdominis plane (TAP) administration) compared to conventional opioids (SMD − 0.56, 95% CI − 1.07, − 0.06, *p* = 0.03). Moreover, LB was associated with decreased length of stay (SMD − 0.34, 95% CI − 0.56, − 0.13, *p* = 0.001) and decreased IV opioid use in the first 48–72 h (SMD − 0.49 95% CI − 0.69, − 0.28, *p* < 0.00001). Byrnes et al. [15] published a network meta-analysis that included 12 trials with a total of 2512 patients undergoing colorectal resections (open or minimally invasive) and demonstrated that LB-based wound infiltration (either local infiltration or TAP administration) reduced morphine usage (mean difference 36.64 mg, 95% credibility interval 15.64–59.20) and length of stay (mean difference 1.79 days, 95% credibility interval 0.59–3.81) compared to standard analgesia (intravenous use of systemic opiates, including infiltration of short-acting local anesthetic). However, after a meta-regression, the findings were only held for minimally invasive surgery. In contrast, other studies did not show a difference in outcomes between LB and short-acting local anesthetic. The recently published TINGLE clinical trial [16] included 102 adults undergoing minimally invasive colorectal surgery with multimodal analgesia. They were randomly assigned to receive a laparoscopic transversus abdominis plane block with liposomal bupivacaine or with bupivacaine with epinephrine and dexamethasone. Their study showed that LB block does not provide superior or extended analgesia in the standardized multimodal analgesia protocols era.

The role of enhanced recovery programs in hemorrhoidectomy is being explored. Chitty et al. [17], in a pre-and post-implementation quality improvement study in patients undergoing hemorrhoidectomy, reported that patient-reported pain scores in the post-anesthesia care unit (PACU) were significantly higher in the bupivacaine compared to the liposomal bupivacaine group (median 3 [IQR 0–6] vs. 0 [IQR 0–4], *p* = 0.03). However, they did not find a difference between opioid rescue and opioid refill requests. Schmidt et al. [18] evaluated analgesia in adults undergoing hemorrhoidectomy, emphasizing the need for a global assessment. They found that comparing LB to the placebo group, the LB group showed a significant reduction in pain intensity at 12–24 h (mean NRS: LB = 2.2, placebo = 2.9, *p* = 0.04) and consumed less opioid rescue medication over 72 h (mean opioids: LB = 10 mg, placebo = 18 mg, *p* = 0.0006).

Our study did not find a difference in adverse effects, like vomiting. Nevertheless, the conventional anesthetic/placebo group reported more pain with bowel movement than the LB group (OR 2.60, 95% CI 1.31–5.16). Knudson et al. [19], in an RCT of 57 patients undergoing elective colon resection, also showed no differences in opiate side effects (anti-nausea medication, return to flatus, or urinary retention) between LB and bupivacaine.

Our study has several strengths. This is the first systematic review and meta-analysis comparing liposomal bupivacaine with placebo or other anesthetics in patients undergoing hemorrhoidectomies. Given the scarce data, this review provides more specific results regarding the use of LB following this type of surgery. We only included randomized, controlled, double-blind clinical trials. Our search was comprehensive, following a systematic methodology, applying pre-specified and detailed data tabulation and extraction and standardized evaluation of evidence quality and publication bias. Multiple researchers rigorously performed all steps. This approach facilitated the identification of a “clean” dataset from comparative studies of different methods to allow better generalizability of the results.

We acknowledge several limitations in our study that are important to address. Firstly, the three studies included had a relatively small number of patients. Additionally, there was large heterogeneity, both in the control group and in dosages of LB.

## Conclusion

Overall, this systematic review and meta-analysis provides valuable insights into the benefits of using liposomal bupivacaine in pain management for hemorrhoidectomy patients. The findings suggest that LB may be useful in reducing postoperative pain and opioid consumption in this specific surgical context compared to short-acting analgesics and placebo.

## Supplementary Information

Below is the link to the electronic supplementary material.

Supplementary file1 (DOCX 36 KB)

## Data Availability

The datasets generated during and/or analyzed during the current study are available at https://github.com/NotLui5/liposomal-bupivacaine_others.
